# Enhanced far infrared emissivity, UV protection and near-infrared shielding of polypropylene composites via incorporation of natural mineral for functional fabric development

**DOI:** 10.1038/s41598-023-49897-2

**Published:** 2023-12-15

**Authors:** Pengfei He, Rayland Jun Yan Low, Stephen Francis Burns, Vitali Lipik, Alfred Iing Yoong Tok

**Affiliations:** 1 School of Materials Science and Engineering, Nanyang Technological University, 50 Nanyang Avenue, Singapore, 639798 Singapore; 2 Physical Education and Sports Science, National Institute of Education, Nanyang Technological University, 1 Nanyang Walk, Singapore, 637616 Singapore

**Keywords:** Materials science, Polymers

## Abstract

Far infrared radiation in the range of 4–20 µm has been showed to have biological and health benefits to the human body. Therefore, incorporating far-infrared emissivity additives into polymers and/or fabrics hold promise for the development of functional textiles. In this study, we incorporated nine types of natural minerals into polypropylene (PP) film and examined their properties to identify potential candidates for functional textiles and apparels. The addition of 2% mineral powders into PP film increased the far-infrared emissivity (5–14 µm) by 7.65%-14.48%. The improvement in far-infrared emissivity within the range of 5–14 µm, which overlaps with the peak range of human skin radiation at 8–14 µm, results in increased absorption efficiency, and have the potential to enhance thermal and biological effects. Moreover, the incorporation of mineral powders in PP films exhibited favorable ultraviolet (UV) protection and near-infrared (NIR) shielding properties. Two films, specifically those containing red ochre and hematite, demonstrated excellent UV protection with a UPF rating of 50+ and blocked 99.92% and 98.73% of UV radiation, respectively. Additionally, they showed 95.2% and 93.2% NIR shielding properties, compared to 54.1% NIR shielding properties of PP blank films. The UV protection and NIR shielding properties offered additional advantages for the utilization of polymer composite with additives in the development of sportswear and other outdoor garments. The incorporation of minerals could absorb near-IR radiation and re-emit them at longer wavelength in the mid-IR region. Furthermore, the incorporation of minerals significantly improved the heat retention of PP films under same heat radiation treatment. Notably, films with red ochre and hematite exhibited a dramatic temperature increase, reaching 2.5 and 3.2 times the temperature increase of PP films under same heat radiation treatment, respectively (46.8 °C and 59.9 °C higher than the temperature increase of 20.9 °C in the PP film). Films with additives also demonstrated lower thermal effusivity than PP blank films, indicating superior heat insulation properties. Therefore, polypropylene films with mineral additives, particularly those containing red ochre and hematite, showed remarkable heat capacity, UV-protection, NIR-shielding properties and enhanced far infrared emissivity, making them promising candidates for the development of functional textiles.

## Introduction

Far infrared radiation (FIR) is an invisible electromagnetic radiation with a wavelength between 3 µm and 1 mm, as defined by the International Commission on Illumination (CIE). According to Kirchhoff's law, the power radiated by an object must be equal to the power absorbed^1^. Research has shown that human skin emits rays in the range of 4–20 µm. Resonance occurs when objects emitting or reflecting radiation within this range are close to the human skin^2^. Therefore, far infrared radiation with the wavelength of 4–20 µm has been demonstrated to offer various health benefits for the human body^3^.

The effect of far infrared radiation could be both thermal and non-thermal. Far infrared radiation transfers energy in the form of heat, which can be perceived by thermoreceptors in the surrounding tissue, and results in thermal effect to increase local temperature. In addition, far infrared radiation could also be absorbed by water in the cells, reducing the size of water clusters and influencing the molecule bonds^4^. Far infrared radiation may also induce a conformational change in the protein level via the receptors on the cell membrane^5,6^. This non-thermal biological effect of far-infrared radiation has been observed in cell and animal studies^7,8^. A human trial conducted in patients undergoing hemodialysis, showed far infrared radiation improved the access flow and survival of the native arteriovenous fistula (AVF) via both thermal and nonthermal effects^9^. The non-thermal biological effects of far infrared radiation offer more potential applications in the healthcare field. In recent times, fabric incorporated with far-infrared emitting additives has emerged as a promising, versatile, and convenient strategy to deliver the health benefits of far infrared radiation in a wide range of applications. Gloves incorporated with far-infrared emitting additives have been shown to increase the temperature of cold hands, activate the thermoregulatory system and enhance blood flow in the capillaries^10^. Similar effects were observed in patients with Raynaud syndrome, a disorder characterized by decreased blood flow to the fingers. Test gloves with FIR emitting additives increased hand temperature and provided pain relief for these patients^11^. FIR belts tested on female subjects with primary dysmenorrhea increased abdominal temperature and increased abdominal blood flow, leading to pain relief^12^. Similar effects, such as pain reduction and improved sleep quality, were observed in patients post-polio syndrome^13^. Furthermore, other benefits such as improved exercise performance and recovery have also been documented^14–16^. Apparels incorporating FIR emitting additives has demonstrated improved performance, shorter exercise recovery from exercise, reduced muscle soreness after exercise^17,18^, decreased oxygen consumption during low-intensity exercise^15^, and less tiredness and relatively stable respiration and heart rate in adults during exercise^19^.

Cell studies and animal studies have explored the potential biological mechanism of far infrared radiation. FIR has been found to potentially stimulate the production of nitric oxide (NO), an important signaling factor that induces vasodilation and increases blood flow^5,7,20,21^. The availability of nitric oxide may further activate certain pathways to trigger other physiological effects, such as mitochondrial biogenesis^22^. This mechanism may explain the observed benefits in the field of sports. Other mechanisms of FIR include anti-inflammatory effects and the reductions of reactive oxygen species (ROS)^23–26^. Volunteers wearing FIR-emitting vests and socks have shown decreased levels of free radicals.

FIR emitting ceramics typically consist of metal oxides such as SiO_2_, Al_2_O_3_ and TiO_2_^12,13,19^. Natural minerals with multiple components such as tourmaline, kaolinite and rhyolite, have been studied for their unique properties^18,27^. These FIR-emitting ceramics and minerals show promise as candidates for thermal management applications, as well as for functional textiles and apparels with various health benefits as abovementioned^28^. Furthermore, inorganic minerals have demonstrated additional benefits, such as antimicrobial properties. Previous studies on socks containing FIR emitting minerals worn by athletes have shown reduced bacterial growth and decreased sweating^29^. The incorporation of mineral particles into polymers to create polymer composite generally presents minimum risk compared to finishing treatment utilizing surface coating methods^30^. Recently, there have been some concerns on the utilization of nanoparticles and their potential impact on wearer’s skin due to their extremely small particle size at the nanoscale^31^. Future studies are necessary to investigate the impacts of nanoparticles on the skin and environments, in order to ensure the safe and responsible utilization of nanoparticles and minimize possible adverse effects.

Inorganic oxides have been identified for their ultraviolet (UV) shielding properties^32^. Specifically, titanium dioxide (TiO_2_) and zinc oxide (ZnO) are widely utilized in sunscreens and approved by the U.S. Food and Drug Administration (FDA)^33^. Cole et al. demonstrated that the UV protection effects of these inorganic UV filters primarily arise from their absorbing ability rather than scattering and reflecting abilities via an optical sampling equipment for measuring the reflection and transmission properties of micron and nano ZnO and TiO_2_ particles^34^. Effective scattering and reflecting visible light as well as absorbing UV radiation in TiO_2_ and ZnO particles are determined by their band gap width of these semiconducting materials. UV absorption occurs when the energy absorption exceeds the band gap width, as elucidated in previous studies on the band gap theory^35–37^. Recently, iron oxides such as hematite have shown UV absorption capabilities. Hoang-Minh et al. prepared various clays and found that the Fe_2_O_3_ content of a clay plays a key role in determining the UV-protection ability. The main reason may be attributed to the empty orbital in the electron configuration of Fe^3+^, similar to the case of TiO_2_, and energy absorption in the near UV leading to a change to a higher energy level^38^. TiO_2_ and ZnO mainly provide UVA and UVB protection, they have limited protection against high-energy visible (HEV) light and near-infrared radiation. Iron oxide has demonstrated effective protection against HEV light between 400 and 500 nm, which significantly contributes to the photoaging of the skin^39^. Safety concerns regarding the cytotoxicity and genotoxicity of TiO_2_ and ZnO particles are increasing recently, primarily attributed to their photocatalytic effects that may generate reactive oxygen species (ROS) upon exposure to UV light. The photocatalytic effects are influenced by the surface properties of these particles, therefore, coatings with other components such as silica are employed as a preventive measure against potential issues^40^. Introducing metal atoms such as Al, Mn, and Na can induce deep bandgap impurity levels, presenting as recombination traps for photogenerated excitons, thus reducing the photocatalytic activity of ZnO^41^. Iron oxide was also demonstrated to have negligible photocatalytic effect in comparison to photocatalytic effect of TiO_2_ and ZnO^42^. Therefore, natural mineral powder containing iron oxide, as well as a combination of metal atoms with photocatalytic oxides such as TiO_2_ and ZnO, may also possess potential UV protection ability.

Considering the potential health benefits associated with FIR emitting minerals, we have incorporated natural minerals microparticles into polypropylene (PP) and investigated the properties of polypropylene films with these additives. In this study, the additives will be incorporated into polymers, the optical properties, thermal properties and heat capacity will be examined. By exploring the properties of different minerals in the PP film, we aim to understand how these minerals behave in composite polymers and identify potential candidates for the development of functional textiles and apparels.

## Materials and methods

### Fabrication of the PP composite film and morphology observations

Eight natural minerals including rose quartz (RQ), red chalcedony (RCD), red jasper (RJP), obsidian (OBS), tourmaline (TM), garnet (GN), hematite (HEM) and magnetite (MGN) were purchased from Mikon Co. (Singapore). In addition, red ochre (ROCH) powders were purchased from Konstantas (https://konstantas.com/). The minerals were milled into powder using a micro powder pulverizer (Tencan GJ-1, China) and sieved using a 10 µm sieve. The composition of the minerals was analyzed by X-ray fluorescence (XRF) (Bruker S8 TIGER, United States). The particle size of the mineral powders was analyzed by a laser diffraction particle size distribution analyzer (HORIBA, Japan). Natural minerals were mixed with polypropylene (LyondellBasell, Netherlands) respectively at a concentration of 2wt% and 4wt% and mixed at 180 °C using a lab twin screw extruder (Wuhan Ruiming, China) under controlled rotational speed. The blended pellets obtained from the extruder were then transferred to a hot press machine for pressing at 180 °C for 10 min using a 150 × 150 × 0.5 mm square mold. A blank sample with sample amount of polypropylene alone was hot pressed using the same conditions and mold. PP composite films were observed under a high-resolution digital microscopy (VHX-7000, Keyence, Japan). The cross-sections of PP composite films were obtained, coated with carbon using a sputter coater (PELCO SC-6, Ted Pella Inc, USA), and observed under a field emission scanning electron microscopy (FE-SEM) with a voltage of 5 kV (JEOL 7600F, Japan).

### FTIR spectra, FIR emissivity, UV-protection and NIR protection properties

The absorbance spectra of blank PP film and films with nine minerals was obtained from 4 to 20 microns using FTIR spectrometer (Perkin Elmer Frontier, USA) with resolution of 4 cm^−1^ and 16 scans through a slide holder with a 1 cm diameter hole. Transmittance (T) was measured in air, and absorbance (Abs) is obtained by using the formula Abs = −logT. Far infrared emissivity of the films was measured during the wavelength range of 5–14 µm at 25 °C using a Far Infrared Emissivity Analysis System (HOTECH EMS302M, Taiwan). The emissivity was measured as a relative value compared to the emissivity of a black body, which was assumed to be 1. The transmission spectra during UV range (280–400 nm) and NIR range (780–1400 nm) were measured by a UV–VIS spectrophotometer (Agilent Cary 60 UV–Vis spectrophotometer, US). The UV protection properties were assessed according to the method described in Australian/New Zealand Standard: Sun protective clothing – Evaluation and classification and previous publication^43,44^. Briefly, the arithmetic means of the UVA transmittance (UVA_AV_), UVB transmittance (UVB_AV_) and ultraviolet protection factor (UPF) were calculated according to the following formulas:

1 $$\mathrm{UVAAV } \, ({\%}) = \frac{{\int }_{315}^{400}T(\lambda )d\lambda }{{\int }_{315}^{400}d\lambda } ({\%})$$

2 $$\mathrm{UVBAV } \, ({\%}) = \frac{{\int }_{290}^{315}T(\lambda )d\lambda }{{\int }_{290}^{315}d\lambda } ({\%})$$

3 $$\mathrm{UPF }= \frac{{\sum }_{290}^{400}E(\lambda )\times S(\lambda )\times \Delta \lambda }{{\sum }_{290}^{400}E(\lambda )\times S(\lambda )\times T(\lambda )\times \Delta \lambda }$$

where T (λ) is spectral transmittance of the sample at wavelength λ. dλ is bandwidth, λ is wavelength in nm, E(λ) is the relative erythemal spectral effectiveness, S(λ) is solar spectra irradiance in W m^−2^ nm^−1^, Δλ is the measured wavelength interval in nm. The higher UPF means higher UV protection. The UPF protection categories are adopted from the standard abovementioned.

The NIR shielding of PP films without and with additives was evaluated by comparing the area under the curve (AUC) of the transmittance spectra with the blank (100% transmittance) and calculated using the following formula:

4 $$\text{NIR shielding } \, ({\%}) = \left(1-\frac{\text{AUC of samples at 780-1400 \, nm or 780-2500\, nm}}{\text{AUC of blank at 780-1400\, nm or 780-2500\, nm}}\right)$$

### Thermal analysis and heat capacity

Thermal analysis by differential scanning calorimetry (DSC) was performed using TA Instruments Q10 DSC. Briefly, approximately 5 mg film samples were heated from 30 to 200 °C at a rate of 10 °C/min, and subsequently cooled to 30 °C at the same cooling rate of 10 °C/min after 1 min. The samples were heated for a second time under the same conditions as those employed during the first heating cycle. Melting temperature (T_m_), crystallization temperature (Tc) and enthalpy change (ΔH) of the samples were then obtained from the second heating process and analyzed. The degree of crystallinity (Xc) was calculated using the following formula (5) as described before^45^:

5 $$Xc=\frac{{\Delta H}_{m}}{\Delta {H}_{m100} (1-{W}_{f})} \times 100\%$$

where *ΔH*_*m*_ is the heat of fusion of the sample, ΔH_m100_ is the heat of fusion of 100% PP polymer, which is 209 J/g. W_f_ is the weight percentage of the additives.

The ability of the films to exchange thermal energy with their surroundings was measured as thermal effusivity using a thermal effusivity meter (Thermtest, Canada) with contact time of 2 s and 10 s at ambient temperature of 24 °C.

The heat capacity of the PP films with additives was measured using an infrared lamp to test the heat storage and release (Refer to Supplementary Fig. S1 for Schematic). The power of the light bulb is 150 watts, and the wavelength range from 0.75 to 5 µm with a peak wavelength of 4 µm. Film samples were placed on a polystyrene plate. The distance of the light and film sample is 30 cm. The duration of the test is 2 min, including 1 min of light on and 1 min of light off. The temperature of the film samples during the 2 min was recorded by an infrared camera (FLIR E86, US) and analyzed by the software FLIR research studio standard.

### Fabrication of polypropylene composite into yarn and tensile strength analysis

Polypropylene composite pellets with 2% hematite or red ochre powders were processed in a twin screw extruder compounder (PSHJ-20, Jiangsu Xinda Tech Limited, China) at the speed of 6.5 kg/h with the temperature profile of five heating zone as 180, 180, 190, 200, 210 °C. Pellets with additives were dried at the oven at 80 °C with dehumidifier overnight before a yarn fabrication.

Multifilament yarn was produced using a melt spinning machine (FET 100, UK). The multifilament yarns were spun using spinneret with 48 holes (0.6 mm each hole size). Temperature profile was set to 200, 210, 220, 220, 240, 250 °C for a screw barrel and 250 °C for a melt pump. Obtained yarn has a linear density of 80 denier. The tensile properties of the yarn were determined using Gester universal testing machine at a gauge length of 250 mm and elongation speed at 300 mm/min, according to standard ASTM D2256. The breaking tenacity (gf/d) was calculated by dividing the breaking force (gf) by linear density of yarn. The percentage of breaking elongation were also calculated.

## Results and discussions

### Composition and particle size of the mineral powders

The nine minerals used in the study are primarily composed of metal oxides, including SiO_2_, Fe_2_O_3_, Al_2_O_3_, CaO, K_2_O, Na_2_O, MgO, P_2_O_5_, BaO, MnO and TiO_2_. Based on their major components as shown in Table 1, the minerals can be categorized into two groups: SiO_2_-rich group and Fe_2_O_3_-rich group. The SiO_2_-rich group includes minerals such as rose quartz (RQ), red chalcedony (RCD), red jasper (RJP), obsidian (OBS), and tourmaline (TM). These minerals contain predominantly SiO_2_, constituting over 70% of the major components. On the other hand, hematite (HEM), magnetite (MGN) and red ochre (ROCH) belong to Fe_2_O_3_-rich group, as they predominantly consist of Fe_2_O_3_. The iron oxide content contributes to the reddish or dark color exhibited by the PP film containing minerals in these samples (Supplementary Fig. S2). Garnet contains both SiO_2_ and Fe_2_O_3_ as its major components.

**Table 1:** Compositions of nine natural minerals.

Composition (%)	RQ	RCD	RJP	OBS	TM	GN	HEM	MGN	ROCH
SiO_2_	98.86	97.65	89.81	79.11	70.74	30.92	2.64	1.97	14.51
Fe_2_O_3_	0.82	1.49	7.40	2.50	2.72	44.96	83.98	83.79	69.34
Al_2_O_3_	0.19	0.43	0.32	9.09	15.47	18.35	1.87	–	10.98
CaO	–	0.15	2.00	–	1.23	–	5.75	–	1.94
K_2_O	–	–	–	4.06	3.19	–	–	–	–
Na_2_O	–	–	–	4.36	2.81	–	–	–	–
MgO	–	–	–	–	2.92	3.46	–	–	–
MnO	–	–	–	–	–	1.29	–	–	–
P_2_O_5_	–	–	–	–	–	–	5.39	–	–
BaO	–	–	–	–	–	–	–	12.62	–
TiO_2_	–	–	–	–	–	–	–	–	1.71

Most of the mineral powders used in the study have a mean particle size of 2–3 µm (Please refer to Supplementary Table S1 for particle size distribution). Red jasper has a slightly larger mean particle size of 4.04 µm, while obsidian and Tourmaline have a particle size of 7.04 µm and 5.73 µm respectively. The incorporation of the microparticles of mineral powder into the polymer to create polymer composite poses minimal risks for human body compared to nanoparticles, due to their large particle size and reduced potential for penetrating into human skin. Most of the micro-sized mineral particles are evenly distributed within the PP film, resulting in a uniform color appearance (Supplementary Figs. S2 and S3a, d). Clusters of larger particles were observed in the films by high resolution digital microscopy and in the cross-section view by SEM imaging (Supplementary Fig. S3).

### FTIR spectra of films with minerals

**Figure 1:**
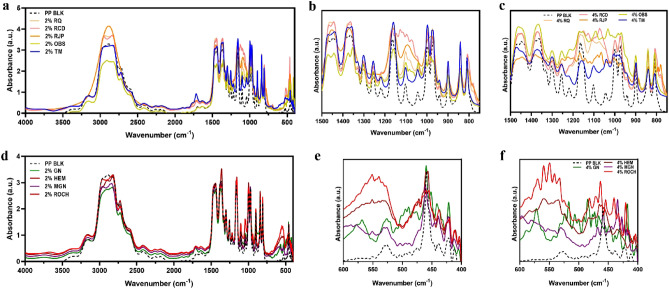
FTIR spectra of film with 2% additives (**a,d**), films with 2% (**b**) and 4% (**c**) SiO_2_-rich minerals at the wavenumber of 750–1500 cm^−1^, and films with 2% (**e**) and 4% (**f**) Fe_2_O_3_-rich minerals at the wavenumber of 400–600 cm^−1^.

FTIR spectra analysis revealed that there were primarily physical bonds between the minerals and the PP films, indicating that the structure of the PP film was not significantly affected by the incorporation of minerals (Fig. 1). All the films exhibited a strong absorbance peak at the wavenumbers of 2830–2970 cm^−1^, which is associated with C–H stretching of the PP polymers. The peaks corresponding to CH_2_ asymmetric stretching peak at 2903 cm^−1^, CH_2_ symmetric stretch peak at 2836 cm^−1^, and CH_3_ asymmetric stretch peak at 2962 cm^−1^ overlapped with each other, resulting in a broad peak (Fig. 1a, d). Films containing additives rich in SiO_2_, such as RQ, RCD, RJP, OBS and TM, showed an increased absorbance over a broad range of peaks at wavenumbers of 1050–1150 cm^−1^, which are associated with Si–O–Si structure present in these minerals (Fig. 1b). Films containing iron oxide-rich minerals including HEM, MGN and ROCH did not exhibit the same trend but showed increased absorbance in the range of 550–600 cm^−1^, which is associated with Fe–O bond. The peak at 551 cm^−1^ was prominent in the HEM film and ROCH film, while the MGN film showed a higher peak at 578 cm^−1^. A similar peak at 578 cm^−1^ was also observed in GN film (Fig. 1e). Previous literature has reported this characteristic peak of Fe–O bond at 548 cm^−1^ and 576 cm^−1^ respectively^46,47^. The peaks corresponding to Si–O–Si and Fe–O bonds became stronger with an increase in the mineral concentration from 2 to 4% (Fig. 1b, c, e, f).

### Far infrared emissivity of the films with minerals

The far infrared emissivity of the films was measured within a wavelength range of 5–14 µm. According to Table 2, the film with the highest emissivity among the 2% additive films was the red ochre (ROCH) film, with an emissivity of 0.959. This represents a 14.55% increase in emissivity compared to the PP blank film. On the other hand, the film with the lowest emissivity was the red chalcedony film, with an emissivity of 0.902, which is a 7.71% increase compared to the PP blank film. Increasing the mineral concentration from 2 to 4% in most of the films further enhanced the FIR emissivity, resulting in a range of 10.7% (0.927 in RQ film) to 17.66% (0.985 in OBS film) higher emissivity than the PP blank films. However, TM and ROCH films maintained their relatively high emissivity and did not show an increase with higher mineral concentration. In general, the incorporation of additives significantly improved the FIR emissivity of the PP films. Far-infrared emitting materials within the 4–20 µm range overlap with the radiation range of the human body and are believed to have health benefits^3^. Far infrared radiation within the range of human body radiation can activate ion channels on cell membranes, stimulating the production of nitric oxide, a signal molecule that can cause muscle relaxation and increased blood flow in smooth muscle cells, and activate a key regulator called peroxisome proliferator-activated receptor gamma coactivator 1-alpha (PGC-1α) to induce mitochondrial biogenesis^5,21,22^. Previous studies have demonstrated the peak radiation of human skin falls within the 8–14 µm range. Yu et al. (2012) measured human skin radiation spectrum at different times of the day and identified two peak regions between 10 and 14 µm^48^. An et al. (2021) found that the human hand exhibits a peak radiation spectrum at 7.5–14 µm^49^. In this study, the enhanced FIR emissivity observed in the films with additives within the range of 5–14 µm measured covers a significant portion of the human body radiation range, including the peak region of human skin radiation. Previous studies have shown that absorption efficiency will be increased when the radiation range of materials emitting or reflecting overlaps with human skin radiation, the resonance and the resulting thermal and biological effects will therefore be enhanced^2,50,51^.

**Table 2:** FIR emissivity at 5–14 µm of the PP films with 2% and 4% additives.

Films	Films with 2% additives	Films with 4% additives
PP BLK	0.838	
RQ	0.922	0.927
RCD	0.902	0.938
RJP	0.911	0.966
OBS	0.949	0.985
TM	0.936	0.935
GN	0.921	0.930
HEM	0.943	0.979
MGN	0.928	0.954
ROCH	0.959	0.959

### UV-protection and NIR-shielding properties

Ultraviolet (UV) and near-infrared (NIR) radiation could be protected by the films and fabric with UV and NIR blocking properties. UV radiation including UVA with wavelength of 315–400 nm and UVB with wavelength of 280–315 nm, are associated with health issues for human body, especially the skin and eyes, sunburn, photoaging, skin cancer, and potentially contribute to some autoimmune and viral diseases^52^. Near infrared radiation with a wavelength of 780–2500 nm could also cause photoaging and bring health issues for the human body, especially IR-A (780 nm to 1400 µm), which could penetrate human skin and reach the dermis. Potential risks also exist with natural solar radiation, which consists of over 30% IR-A^53^. Use of therapeutic or wellness equipment with artificial IR-A sources with high irradiance for a prolonged exposure could increase the risks of health problems^54^.

**Figure 2:**
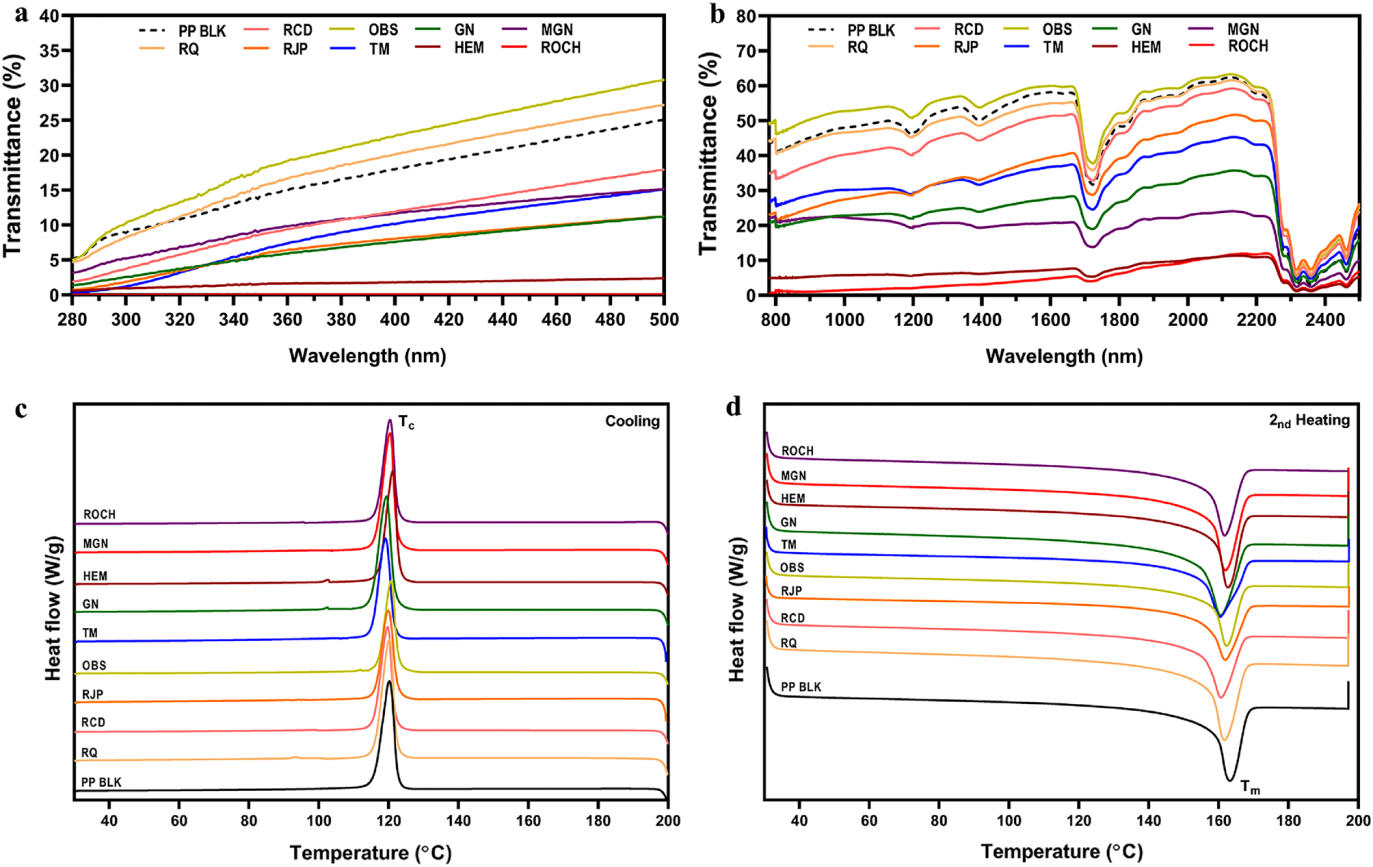
UV–vis and DSC characterization of PP films with 2% additives. (**a**) UV–vis spectra during UV radiation of 280–400 nm and HEV radiation of 400-500 nm. (**b**) UV–vis spectra during NIR radiation of 780–2500 nm. (**c**) DSC curve of PP films with 2% additives during cooling process. (**d**) DSC curve of PP films with 2% additives during 2nd heating process.

Table 3 demonstrates the UV protection properties of the films and UV–vis spectra was shown in Fig. 2a. The HEM and ROCH films exhibited excellent UV protection, with a UPF (Ultraviolet Protection Factor) value of 50+ (UPF 68.66 and 506.31). These films blocked 98.73% and 99.92% of ultraviolet radiation (UVR), specifically 98.45% and 99.9% of UVA, and 99.01% and 99.94% of UVB. RJP, TM and GN films also showed good UV protection, with UPF values of 19.57, 17.37 and 20.42 respectively. The other additives did not exhibit UV protection properties. Specifically, ROCH and HEM films demonstrated the ability to block high-energy visible (HEV) light within the range of 400–500 nm, as shown in Fig. 2a.

**Table 3:** Mean ultraviolet protection factor (UPF), UPF protection category, mean UVA, UVB and ultraviolet radiation (UVR) transmittance of the films with additives.

Films	UPF	UPF protection category	UVA_AV_ (%)	UVB_AV_ (%)	UVR_AV_ (%)
PP BLK	7.99	No protection	14.51	9.21	11.86
RQ	7.34	No protection	15.94	8.56	12.25
RCD	13.05	No protection	8.98	4.02	6.50
RJP	19.57	Good protection	5.97	2.10	4.04
OBS	6.34	No protection	18.33	10.61	14.47
TM	17.37	Good protection	6.88	1.50	4.19
GN	20.42	Good protection	5.71	2.72	4.21
HEM	68.66	Excellent protection	1.55	0.99	1.27
MGN	12.28	No protection	9.42	5.41	7.41
ROCH	506.31	Excellent protection	0.10	0.06	0.08

Commonly used sunscreen lotions may effectively block UV radiation, but may not offer sufficient protection against near-infrared radiation. However, the films with mineral additives in the study showed significant NIR shielding properties in addition to UV protection (Fig. 2b). The area under the curve (AUC) at the wavelength of 780–2500 nm was compared to that of PP film without additives. The ROCH and HEM films exhibit NIR shielding of 93.2% and 95.2% respectively, representing a 72.3% and 76% increase compared to the PP blank film (54.1%). MGN and GN film also showed NIR shielding properties of 81.1% and 76%, respectively (Table 4).

**Table 4:** The area under the curve (AUC) at 780–2500 nm of the transmittance spectra and the NIR shielding properties of the PP films with additives.

Samples	AUC_780–2500 nm_	NIR shielding (%)
Blank (100% transmittance)	172,100.0	
PP blank	78,981.8	54.1
RQ	78,811.2	54.2
RCD	72,147.7	58.1
RJP	57,565.1	66.6
OBS	84,989.8	50.6
TM	53,745.1	68.8
GN	41,380.0	76.0
HEM	11,618.7	93.2
MGN	32,442.5	81.1
ROCH	8296.4	95.2

### Thermal properties by DSC analysis

The melting endotherm of PP film with additives was observed by DSC analysis (Fig. 2c, d) and summarized in Table 5. The addition of mineral additives resulted in an increased degree of crystallinity in the PP films, indicating that the minerals enhanced the nucleation process during the film formation. In terms of the melting temperature, a slight decrease was observed in the PP films with mineral additives compared to the PP blank film. Specifically, the TM film showed the lowest melting temperature among the different mineral additives. This reduced melting temperature of polymers with tourmaline microparticles was also observed in previous publications^55^.

**Table 5:** DSC melting temperatures (T_m_), crystallization temperature (Tc), enthalpies of melting (ΔH_m_) and degree of crystallinity (X_c_) of PP films with 2% additives.

	T_m_ (°C)	T_c_ (°C)	ΔH_m_ (J/g)	Xc (%)
PP BLK	163.23 ± 0.05	120.41 ± 0.29	78.24 ± 1.05	37.44 ± 0.5%
RQ	161.63 ± 0.00	120.20 ± 0.08	84.02 ± 2.02	41.02 ± 0.98%
RCD	160.37 ± 0.45	119.54 ± 0.21	85.50 ± 2.50	41.74 ± 1.22%
RJP	161.13 ± 1.10	119.69 ± 0.28	81.45 ± 0.48	39.77 ± 0.23%
OBS	162.33 ± 0.04	120.74 ± 0.08	89.07 ± 1.58	43.49 ± 0.77%
TM	159.83 ± 0.64	118.88 ± 0.29	78.39 ± 0.55	38.27 ± 0.27%
GN	160.45 ± 0.24	119.35 ± 0.01	86.56 ± 3.42	42.26 ± 1.67%
HEM	162.38 ± 0.38	120.85 ± 0.47	95.62 ± 3.96	46.68 ± 1.93%
MGN	162.61 ± 1.03	120.18 ± 0.27	81.37 ± 1.29	39.73 ± 0.63%
ROCH	162.35 ± 0.91	120.69 ± 0.39	81.28 ± 3.22	39.68 ± 1.57%

### Heat retention capacity of PP films with additives

**Figure 3:**
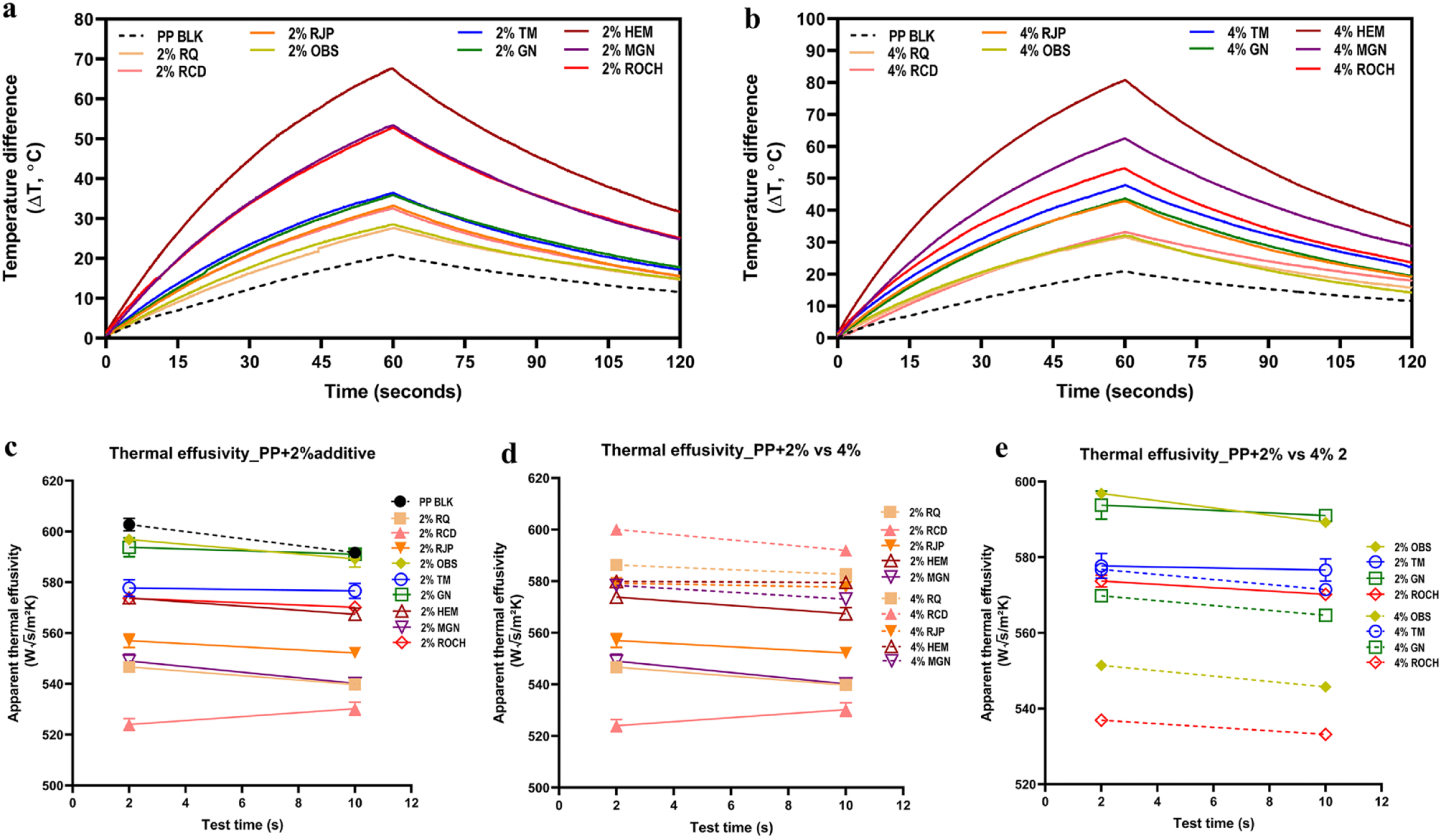
Heat retention capacity and thermal effusivity of PP films with additives. Change of temperature difference from the initial temperature of films with 2% additives (**a**) and 4% additives (**b**) was observed under infrared light for 1 min (0–60 s) and 1 min after infrared light off (60–120 s). (**c**) Thermal effusivity of PP films with 2% additives at 2 s and 10 s touching time. (**d**) Films with additives showing increased thermal effusivity with the increase of concentration from 2 to 4%. (**e**) Films with additives showing decreased thermal effusivity with the increase of concentration from 2 to 4%.

The thermal effects and heat capacity of the PP films with mineral additives were evaluated using an infrared light source and continuous measurement with an infrared camera. The films were treated with the same heat radiation, however, they showed varied temperature increases depending on their heat capacity. The results demonstrated that all films with 2% and 4% additives exhibited enhanced heat capacity compared to the PP blank film (Fig. 3 and Table 6). Generally, additives rich in SiO_2_ showed lower temperature increases compared to the additive rich in Fe_2_O_3_. Hematite film demonstrated the highest heat capacity, with temperature increases of 67.7 °C in 2% HEM film and 80.8 °C in 4% HEM film after 1 min of infrared light radiation. These values were 46.8 °C and 59.9 °C higher than the temperature increase of 20.9 °C in the PP blank film respectively. Magnetite and red ochre film also exhibited significant temperature increase, with MGN film showing a temperature increase of 53.4 °C in 2% film and 62.5 °C in 4% film, and ROCH film showing a temperature increase of 52.8 °C in 2% film and 53.1 °C in 4% film respectively. Even after a 1-min cooling period, HEM, MGN and ROCH films still exhibited higher temperature increases compared to the PP blank film. The temperature increase for these films ranged from 23.6 to 34.9 °C, which was significantly higher than the temperature increase of 11.6 °C observed in the PP blank film. This indicates that HEM, MGN, and ROCH films retained more heat even after the heat source was removed, demonstrating their superior heat retention capacity. Although RQ film and OBS film showed the lowest heat capacity among all the additives, they still exhibited enhanced heat capacity compared to PP blank films.

**Table 6:** Highest temperature difference (ΔT_max_) at 1 min under infrared light and final temperature (ΔT_2min_) at 1 min after infrared light is off.

Minerals additives	Films with 2% additives		Films with 4% additives	
	Highest temperature difference (ΔT_max,_ °C)	Final temperature difference (ΔT_2min_, °C)	Highest temperature difference (ΔT_max_, °C)	Final temperature difference (ΔT_2min_, °C)
PP BLK	20.88	11.62	20.88	11.62
RQ	27.62	14.72	31.61	15.77
RCD	32.54	15.53	33.19	17.88
RJP	33.22	15.51	42.93	19.13
OBS	28.52	14.82	32.10	14.17
TM	36.46	17.18	47.86	22.16
GN	35.90	17.76	43.71	19.39
HEM	67.67	31.67	80.76	34.85
MGN	53.41	24.84	62.52	28.83
ROCH	52.82	25.07	53.11	23.61

The thermal effects of the additives can be attributed to the increased absorbance of the additives within this wavelength of 0.75–4 µm. As mentioned in the UV–vis spectrum analysis, there was also increased absorbance during 780–2500 nm, which covers the near-infrared region and some of the mid-infrared region. Anderson et al. have investigated the optical effects of the fabric with and without ceramic additives in the wavenumber from 3000–12,000 cm^−1^ and reported higher absorbance in fabrics with ceramic^56^. They also observed a spectral shift in the incident radiation, indicating that fabrics with ceramic could absorb near-infrared radiation and re-emit at longer wavelength in the mid-infrared region of 2.5–16.7 µm. This region overlaps with the human body radiation region of 4–20 µm. The property of spectral shift in incident radiation, along with the remarkable heat capacity observed in this study, suggests that the polymer films with mineral additives hold potential for applications in functional textiles for the human body.

### Thermal effusivity of PP films with additives

Thermal effusivity is a thermal property that characterizes the ability of a material to exchange thermal energy with its surrounding environment. Higher effusivity indicates a material's capacity to transfer heat more effectively, making it feel cold to the touch when at the same temperature as the surroundings. Conversely, lower effusivity results in a warmer sensation as the material has a reduced capability to exchange heat. In general, the PP film with 2% additives and 4% additives showed similar or slightly lower thermal effusivity at 2 s and 10 s touch times, indicating a slight “warmer” feeling upon touch (Fig. 3c). Among the films, 2% RQ film showed the lowest effusivity of 524 W S^1/2^ m^−2^ K^−1^ at 2 s touch times and 530.2 W S^1/2^ m^−2^ K^−1^ at 10 s touch times. This decrease in thermal effusivity may indicate the increased thermal insulation properties after the incorporation of mineral additives. Furthermore, the effects of concentration on thermal effusivity varied among different films. Increased concentration from 2 to 4% generally resulted in higher thermal effusivity for films such as RQ, RCD, RJP, HEM and MGN film (Fig. 3d). However, increased concentration from 2 to 4% resulted in lower thermal effusivity in films such as OBS, GN and ROCH film (Fig. 3e). For films containing tourmaline (TM), there was no significant effect of concentration on thermal effusivity, with both 2% and 4% films showing similar values at 2 s touch time and slight differences at 10 s touch time. The impact of mineral concentration on thermal effusivity can vary among different mineral types.

### Application of PP composites with mineral powders in yarn and fabric

In the study, polypropylene composites incorporating nine different minerals were examined. Hematite and red ochre were then selected for the fabrication of yarn and fabric due to their exceptional thermal properties, UV and NIR protection properties compared to the other minerals. 2% of hematite and red ochre were fabricated into yarn with a yarn density of 80 denier. The mineral content was maintained within 2% to enable the spinning of the yarns. We checked the mechanical properties of the yarns and the results were showed in Supplementary Fig. S4. The breaking tenacity of PP blank yarn was 5.20 ± 0.33 gf/day, while PP yarn with 2% hematite (HEM yarn) has an equivalent breaking tenacity at 5.04 ± 0.27 gf/day. PP yarn with 2% red ochre (ROCH yarn) has a slight lower breaking tenacity at 3.99 ± 0.33 gf/day. The breaking elongation for PP, HEM and ROCH yarn are 49.76 ± 4.09%, 57.19 ± 3.96% and 46.73 ± 4.19% respectively. The addition of the mineral particles will influence the microfibrillar structure including crystalline blocks and amorphous crystals, which is also influenced by the parameters such as melt viscosity and drawing speed during processing^57^. In our study, the addition of 2% hematite and red ochre did not disrupt the spinnability of polypropylene yarn, allowing for development of knitted fabric without any difficulties. The images of knitted polypropylene fabric with 2% hematite and red ochre were shown in Supplementary Fig. S4. Our fabricated yarns are partially oriented yarn (POY) and meet the typical tenacity requirement for POY in industry standard (minimum 2 gf/day)^58^. Further processing and optimizations may enhance the breaking tenacity to suit various applications. Delving deeper into the properties of the yarn goes beyond the scope of the present study and will be addressed in future research. Moreover, detailed characterizations regarding the fabric, as well as the exploration on the functionality and their impact on the human body in human trials will also be investigated in subsequent studies.

## Conclusions

In this study, polypropylene films incorporating nine types of natural mineral additives were produced, and their optical properties and thermal properties were investigated. The addition of 2% and 4% additives led to improvement in PP films, including enhanced far-infrared emissivity within 5–14 µm range, superior UV protection, NIR-shielding properties, and increased thermal capacity. The extent of improvement varied depending on the specific mineral additive used. Of all the mineral additives, red ochre and hematite demonstrated the most significant effects. They exhibited high far infrared emissivity, with values of 0.959 and 0.943 respectively, representing a 14.55% and 12.5% increase compared to the PP blank film’s emissivity of 0.838. Additionally, PP films incorporating red ochre and hematite displayed excellent UV protection properties, achieving a UPF 50+ rating. Furthermore, these films showed the highest increase of 95.2% and 93.2% NIR-shielding properties in the 780–2500 nm wavelength range respectively. The incorporation of red ochre and hematite also significantly improved the heat capacity of PP films. These films exhibited temperature increases that were 2.5 and 3.2 times the temperature increase of PP films under the same heat radiation treatment, indicating a remarkable enhancement in heat capacity. The outstanding heat capacity, UV-blocking and NIR-blocking properties, and superior far infrared emissivity observed in the PP films with mineral additives make them promising candidates for the development of functional apparels and textiles with enhanced performance and potential health benefits.

### Supplementary Information

Supplementary Information.

## Data Availability

The authors declare that the data supporting the findings of this study are available within the paper and its Supplementary Information files. Should any raw data files be needed in another format they are available from the corresponding author upon reasonable request.
