# Supplementary material for: Enhanced far infrared emissivity, UV protection and near-infrared shielding of polypropylene composites via incorporation of natural mineral for functional fabric development
Source: Sci Rep. 2023 Dec 15;13:22329. doi: 10.1038/s41598-023-49897-2 (PMC10724279; doi:10.1038/s41598-023-49897-2)

**Supplementary Figure** **S1**: Schematic showing the experimental setting to study the heat capacity of the polypropylene films with mineral powders.


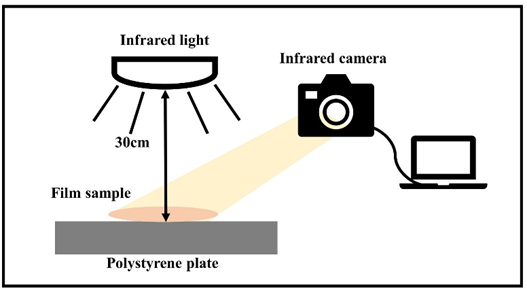


**Supplementary Table S1**: Particle size analysis with distribution size (d10, d50 and d90) of the mineral powders.

| Minerals | d_10_ (µm) | d_50_ (µm) | d_90_ (µm) |
| --- | --- | --- | --- |
| RQ | 1.08 | 3.02 | 5.52 |
| RCD | 0.75 | 2.72 | 5.26 |
| RJP | 1.84 | 4.04 | 6.69 |
| OBS | 2.24 | 7.04 | 14.35 |
| TM | 2.00 | 5.73 | 11.02 |
| GN | 0.68 | 2.58 | 4.87 |
| HEM | 0.52 | 2.50 | 5.39 |
| MGN | 0.56 | 2.43 | 4.86 |
| ROCH | 0.20 | 2.60 | 6.72 |
|  |  |  |  |

**Supplementary Figure S2:** Photos showing polypropylene film (PP BLK) and PP films with 2% and 4% mineral powder.


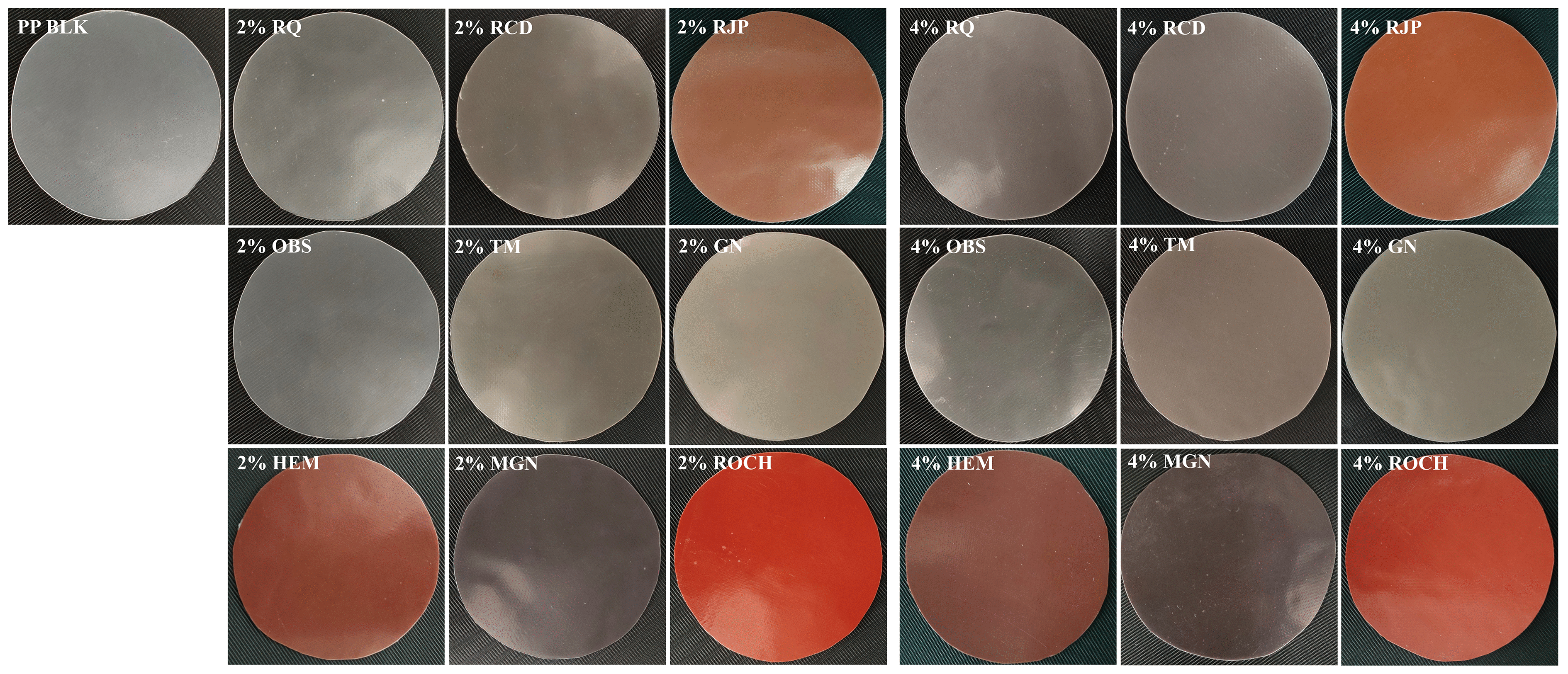


**Supplementary Figure S3:** Morphology observation of PP films observed by high-resolution digital microscopy (**a** and **d**) and SEM (**b**, **c**, **e** and **f**). **a** PP films with 2% hematite particles observed by digital microscopy. **b** and **c** SEM observation of cross-section of PP films with 2% (**b**) and 4% (**c**) hematite particles. **d** PP films with 2% red ochre particles observed by optical microscopy. **e** and **f** SEM observation of cross-section of PP films with 2% (**e**) and 4% (**f**) red ochre particles. Scale bar = 50 µm for **a** and **d**. Scale bar = 10 µm for **b**, **c**, **e** and **f**.


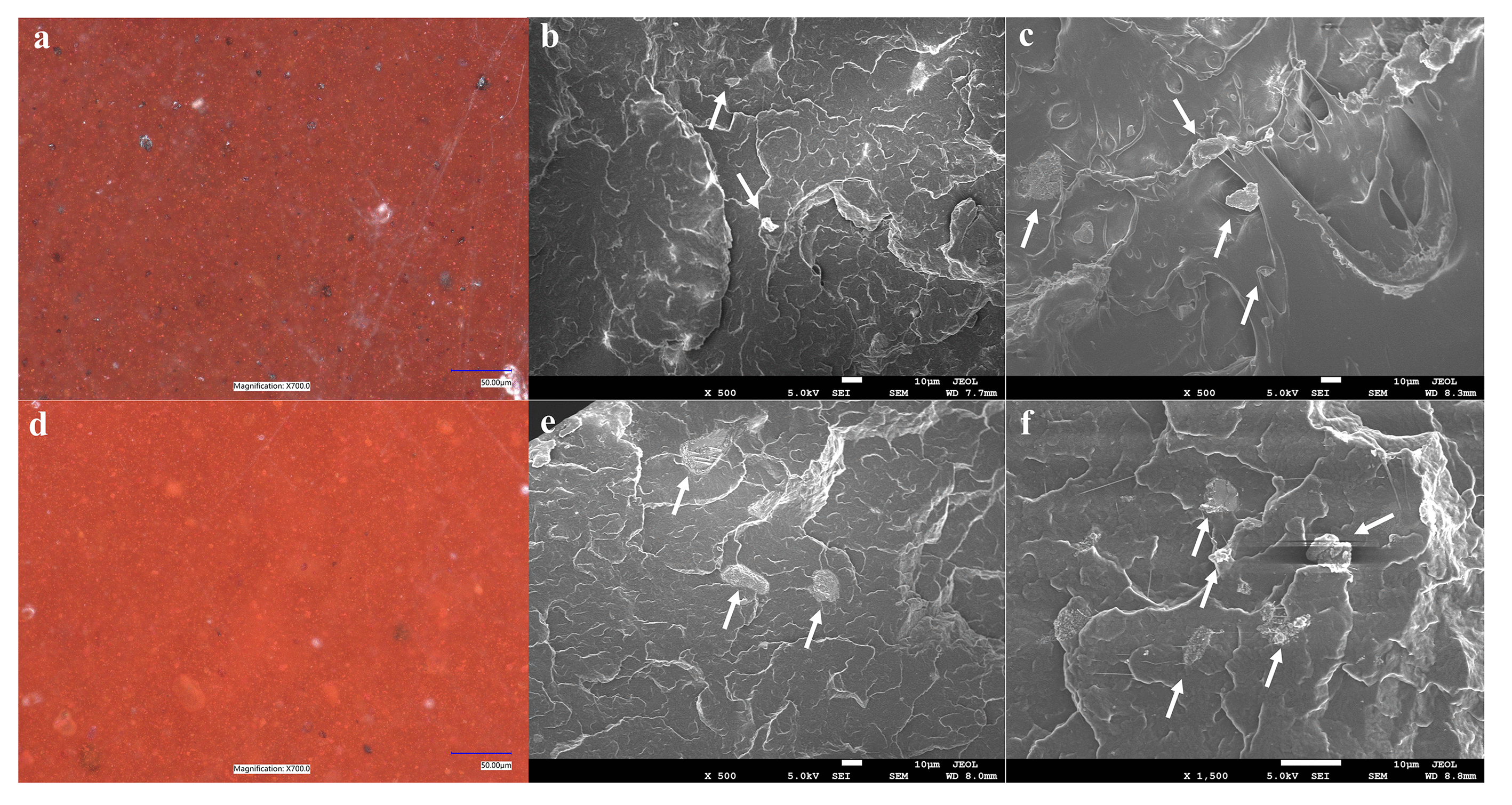


**Supplementary Figure S4:** Tensile strength test of the yarn and images of knitted fabric with minerals powders. **a** Breaking Tenacity of the yarns with a linear density of 80 denier (n=8). **b** Elongation at breaking of the yarns (n=8). **c** polypropylene fabric with 2% hematite particles. **d** polypropylene fabric with 2% red ochre particles.


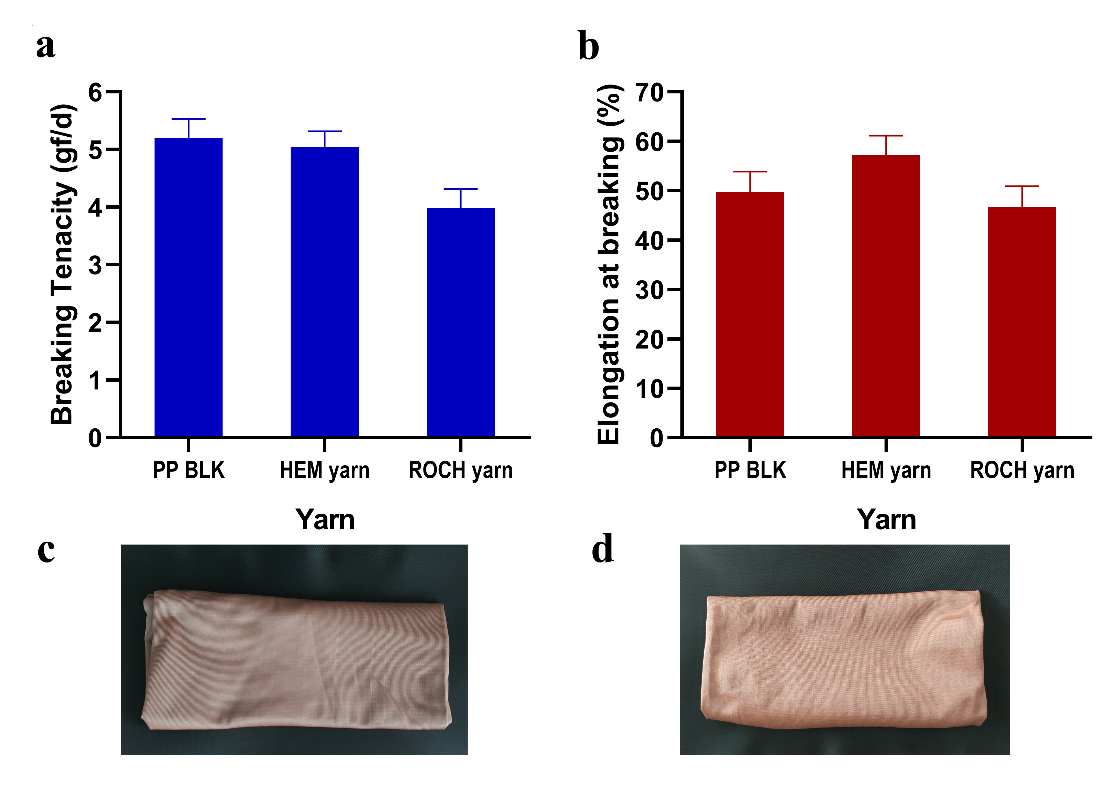

Supplement: Supplementary file 1 — Supplementary Information. [file 41598_2023_49897_MOESM1_ESM.docx]
